# Self-care Behaviors and Technology Used During COVID-19: Systematic Review

**DOI:** 10.2196/35173

**Published:** 2022-06-21

**Authors:** Fareeya Sakur, Kanesha Ward, Neha Nafees Khatri, Annie Y S Lau

**Affiliations:** 1 Australian Institute of Health Innovation Macquarie University North Ryde Australia

**Keywords:** self-care, self-management, chronic conditions, COVID-19, pandemic, technology, digital health, telehealth, health technology

## Abstract

**Background:**

Self-care behaviors are essential for people living with chronic conditions; however, the outbreak of the COVID-19 pandemic has imposed additional complications on their daily routines. Few studies have analyzed how self-care behaviors have changed during COVID-19 and the role of digital technology, especially among people with chronic conditions.

**Objective:**

This study aims to review how self-care behaviors have changed for people with chronic conditions during the COVID-19 pandemic, and what technology they have adopted to manage their conditions during that period.

**Methods:**

A systematic review was conducted using narrative synthesis. Data were extracted from PubMed, MEDLINE, EMBASE, PsycINFO, CINAHL, and Google Scholar, including articles from December 2019 onward. Eligible studies focused on adults diagnosed with chronic conditions undertaking any self-care tasks in line with the middle-range theory of self-care of chronic illness (ie, self-care maintenance, monitoring, and management). The methodological quality of the included articles was assessed with the McMaster Critical Review Forms for Quantitative or Qualitative Studies.

**Results:**

In total, 36 primary research articles were included. Changes to self-care behaviors during COVID-19 among people with chronic conditions were organized according to the middle-range theory of self-care of chronic illness focusing on self-care maintenance (ie, medication adherence, physical activity, and diet control), self-care monitoring (ie, monitoring signs and symptoms), and self-care management (ie, consultations with health care providers). Positive self-care behaviors observed include the following: individuals trying to maintain good glycemic control during COVID-19 increased their medication adherence in 27% (10/36) of studies; and diet control improved in 50% (18/36) of studies. Negative self-care behaviors observed include the following: decline in physical activities and increased sedentariness were observed in 65% (23/36) of studies; poor diet control was observed in 57% (21/36) of studies; and self-monitoring of health status dropped in 43% (15/36) of studies. The use of technology to support self-care of chronic conditions during COVID-19 was reported in 72% (26/36) of studies. The actual use of telehealth in place of physical consultations during COVID-19 was observed in 50% (18/36) of studies, and other digital technologies (eg, social media apps, smartphone apps, web-based platforms, and web browsing) were used in 50% (18/36) of studies. Telehealth was discussed and recommended as the default technology in delivering future health care services during COVID-19 and beyond in 77% (28/36) of studies.

**Conclusions:**

This review highlighted the necessity to rethink how models of self-care should continue to address the demands of chronic conditions while being responsive to the imminent threats of infectious diseases. Perhaps the silver lining of COVID-19 is that adoption of digital technology (especially telehealth) among a vast cross-section of people with chronic conditions is possible. Future research should investigate effective ways to incorporate evidence-based digital health tools into these new models of self-care that address the challenges of chronic and infectious conditions.

## Introduction

### Background

Chronic conditions are an ongoing cause of disability, ill health, and premature death worldwide, and the World Health Organization defines chronic conditions as conditions that are noncommunicable and are of long duration and slow progression [1]. Self-care is essential for people with chronic conditions in order to maintain good control of their health [2]. People with chronic conditions need continuity of care to ensure their conditions are well maintained [3]. However, the public health response to the pandemic (eg, lockdown measures and social distancing) has significantly disrupted this continuity and thus affected people with chronic conditions [4-7].

People with major chronic conditions are not only at a higher risk of COVID-19 infection, but also of worsening their chronic disease outcomes during the pandemic [6-8]. Clinical studies in the United States and Italy undertaken on patients with COVID-19 found that the severity rates and death rates among patients with underlying chronic conditions were 7 times higher than those of patients with nonchronic conditions [8]. From an individual perspective, self-care behaviors have been significantly affected during the pandemic [4,5]. People have had their in-person health care appointments converted to teleconsultations [9]. Many have experienced disruptions in their medication supplies [10]; had limited access to investigative tests (eg, blood tests) [11]; were confronted with barriers to physical activities (PA) [12], imbalanced diets (eg, disruptions in access to food sources) [13], as well as disrupted routines and supplies to necessities; and many have experienced social isolation (eg, not being able to see family and friends), anxiety, and mental distress [12].

The impact of the pandemic on self-care behaviors of people living with chronic conditions was assessed in 2 rapid reviews conducted by Kendzerska et al [12] and Hartmann-Boyce and Mahtani [14] in 2020.

To our knowledge, systematic review–based evidence on how chronic disease self-care behaviors have changed during COVID-19, and how people with chronic conditions have adopted the use of technological aids during COVID-19 to sustain their self-care behaviors remains lacking.

### Objectives

In this study, we will undertake a systematic review to examine how self-care behaviors among people with chronic conditions have changed during COVID-19, and the role of digital technology in facilitating those changes. The research questions in this systematic review are as follows: “How have self-care behaviors among people with chronic conditions changed as a result of COVID-19” and “What technological aids have people with chronic conditions used (or adopted) for self-care during COVID-19?”

## Methods

### Materials and Methods

This systematic review was registered in the International Prospective Register of Systematic Reviews (PROSPERO) with the registration number CRD42021274000.

The review is in compliance with the PRISMA (Preferred Reporting Items for Systematic Reviews and Meta-Analyses) statement [15]. Details of the PRISMA checklist can be found in Multimedia Appendix 1.

### Search Strategy

A modified population, interventions, comparisons, and outcomes (“PICO”) strategy was used to search, with “Population” corresponding to “people with chronic conditions” and “Intervention” as the “self-management of chronic conditions (and the use of technological aids) during COVID-19”; “Comparison” is described as “self-management (and the use of technological aids) before COVID-19,” and “Outcomes” are “changes in self-care behaviors and the use of technology.”

A search from March 6, 2021, to March 11, 2021, was conducted in PubMed, MEDLINE, EMBASE, PsycINFO, CINAHL, and Google Scholar, including all articles published from December 2019. Search terms were designed to capture publications on people living with chronic conditions, their self-care behaviors during COVID-19, and any use of technological aids. Multimedia Appendix 2 provides the complete search strategy.

### Inclusion and Exclusion Criteria

Articles were eligible if they had the following criteria: focused on adults diagnosed with chronic conditions (conditions that limits self-care, requires medical interventions, and lasts more than 6 months); included a quantitative or qualitative component; focused on reporting self-care tasks during COVID-19, undertaken by people diagnosed with chronic conditions; included use of technology in self-care of chronic conditions; and were published in the English language from December 2019.

Articles were excluded if they had the following: did not focus on people with chronic conditions (eg, caregivers or care providers); were not COVID-19–related; focused on purely educational programs to improve self-management of chronic conditions; focused on technology only with no outcome measures; and were protocol papers or opinion articles.

Multimedia Appendix 3 provides the complete criteria.

### Study Screening

Full details on abstracts, full-text screening, and data extraction are provided in Multimedia Appendix 4. Each abstract was screened independently by 3 reviewers, disagreements were resolved by consensus, and full-text screening was undertaken by 1 reviewer.

Data extraction was led by 1 reviewer, and a narrative synthesis was conducted to synthesize the findings of the studies. The 36 included articles were read in full, and specific details on self-care behaviors were extracted and organized into the themes of physical control, medication adherence, diet control, monitoring health status, and consultations with health care providers in a tabular form. Specific items on use and recommendation of technology were extracted and summarized in a tabular form and presented in appendices.

### Methodological Quality Assessment

The McMaster Critical Appraisal Tools for Quantitative Studies and Qualitative Studies was used [16]. Each individual component is rated as “yes,” “no,” “not addressed,” or “not applicable.” A score of 1 was given to “yes,” 0 to “no” and “not addressed,” while items rated as “not applicable” were removed from the total score. Quantitative studies were assessed over 8 main components of study purpose, literature review, study design, sample, outcomes, intervention, results, and conclusions—with the maximum total score being 14. Qualitative studies were assessed over 8 components, which were study purpose, literature review, study design, sampling, data collection, data analysis, overall rigor, and conclusions—with the maximum total score being 22. Methodological quality score rating did not warrant exclusion of studies. The results of the assessment of methodological quality are outlined in Multimedia Appendix 5.

### Theoretical Framework

**Figure 1:**
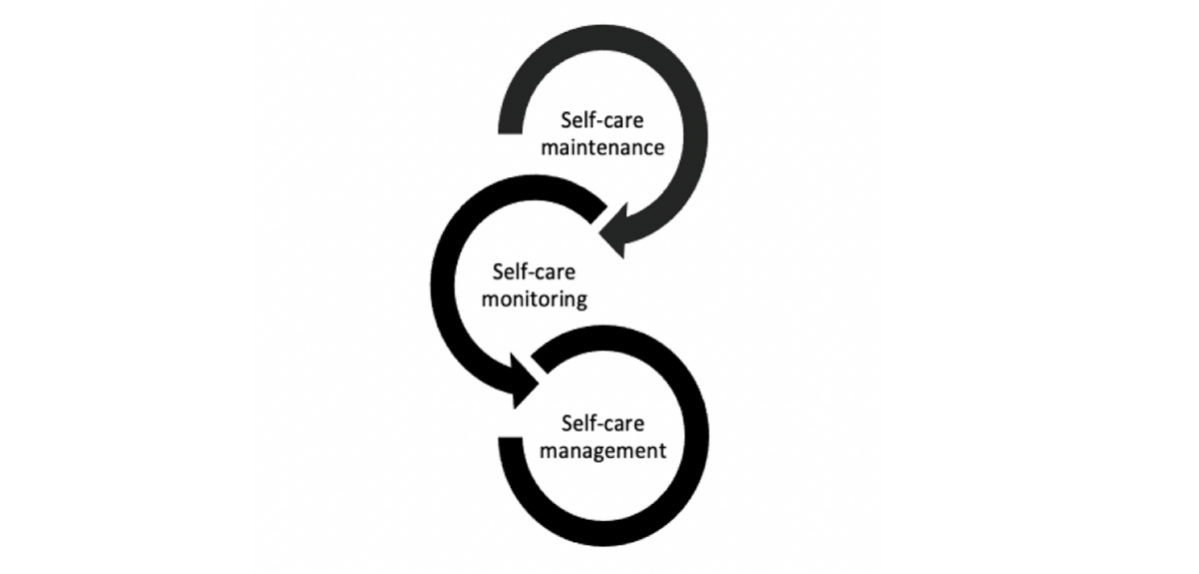
Middle-range theory of self-care of chronic illness model based on three key concepts of self-care behavior [2].

The changes in self-care during COVID-19 and the technology used by people living with chronic conditions were reported according to the middle-range theory of self-care of chronic illness. This theory arose from clinical experience caring for persons with heart failure in 2012 [2]. Self-care is described as the maintenance of health. It is a process undertaken through health promotion practices and management of health conditions that can be performed in a healthy or ill state [2]. The focus is on the following three key concepts: self-care maintenance, self-care monitoring, and self-care management (Figure 1) [2]. The operational definitions and examples of the three key concepts are outlined in Table 1 [2,17].

**Table 1:** The operational definitions and examples of the three key concepts of the middle-range theory of self-care for chronic illness.

Self-care behaviors	Definition	Examples
Self-care maintenance	Behaviors adopted by people to maintain physical and emotional stability for their chronic conditions. They can be strategies set by the individuals alone or based upon recommendations determined between individuals and their health care providers. These behaviors can be related to lifestyle such as diet control, physical exercise, and taking prescribed medications [2,17].	Combining 15 min of postmeal walking with 30 min of resistance training.
Self-care monitoring	A process that involves routinely observing for changes in signs and symptoms with vigilance and acting accordingly [2,17]. It encompasses systematic and routine monitoring. Individuals that are skilled in monitoring their symptoms and communicating them to their health support team help produce the best health outcomes [2,17].	Checking their blood glucose levels daily.
Self-care management	Evaluating changes in signs and symptoms (from both emotional and physical well-being perspectives) that are present due to sickness, treatments undertaken, or the environment. If a response is needed, then a treatment plan can be sought, implemented, and evaluated. The efficacy of the treatment plan in achieving the desired health outcomes is assessed on an ongoing basis, between the individual and their health care team [2,17].	During monitoring, if blood glucose levels are elevated, then a treatment plan can be set in consultation with their health care provider.

## Results

### Screening Process

The database search retrieved 498 publications, and 122 duplicates were removed. After title and abstract screening, 289 publications were removed. Search updates led to 9 publications being included in the screening. After full text screening, 63 publications were excluded, leaving 33 included articles. A further 3 articles were identified by searching the reference lists of the included articles. The entire screening process concluded with the inclusion of 36 original research publications.

**Figure 2:**
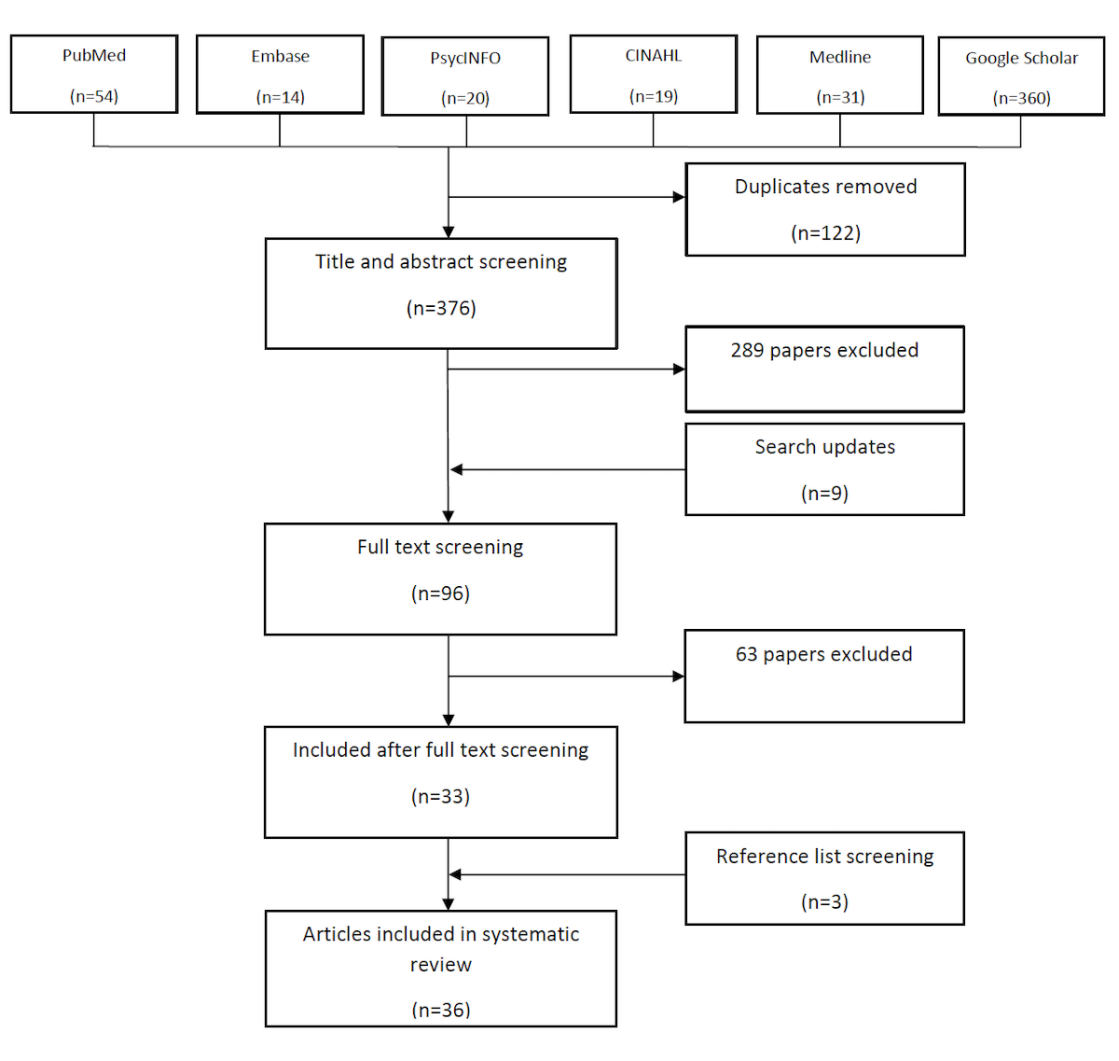
Number of articles included at each stage of the screening process.

The literature selection process is outlined in Figure 2. Multimedia Appendix 4 provides more details about the screening process.

### Characteristics of the Included Articles

A total of 36 original research publications were included in the systematic review. The most common research strategy employed was semistructured online interviews. The majority of studies were conducted in India (10/36, 28%), followed by United Kingdom (5/36, 14%); United States and Brazil (4/36, 11%); Spain (3/36, 8%); Hong Kong and Arab states (2/36, 6%); and Pakistan, Israel, Denmark, Poland, Canada, Saudi Arabia, and Australia (1/36, 3%).

Over 28 health conditions were studied in the included articles: diabetes mellitus (26/36, 72%), hypertension (8/36, 22%), cancer (7/36, 19%), cardiovascular diseases (6/36, 17%), chronic heart diseases (6/36, 17%), respiratory conditions (6/36, 17%), chronic kidney diseases (5/36, 17%), and chronic obstructive pulmonary disease (4/36, 11%).

The different health conditions included in the articles are further outlined in Multimedia Appendix 6. The conditions most frequently stated in the included studies are diabetes mellitus (26/36, 72%), hypertension (8/36, 22%), and cancer (7/36, 19%).

The self-care behaviors included in the articles are provided in Multimedia Appendix 7. Instances where technology was stated in the included articles are outlined in Multimedia Appendix 8. Additionally, Multimedia Appendices 9 and 10 provide more information on the included articles.

### Self-care Behaviors Adopted by Participants

People living with chronic conditions are embodied with the responsibility for their daily care and actively engage in tasks essential for long-term management of their conditions [2,17].

Self-care behaviors observed in the included studies are organized according to the middle-range theory of self-care of chronic illness. These include self-care maintenance (ie, medication adherence, physical activity, and diet control), self-care monitoring (ie, monitoring signs and symptoms), and self-care management (ie, consultations with health care providers). The technology reported in these studies was organized according to the technology used for self-care during COVID-19 and technology recommended for self-care during and beyond COVID-19. The results of self-care behaviors found in the included articles are illustrated in Table 2.

**Table 2:** Results of self-care behaviors in the included articles (N=36).

Self-care behaviors		Increase reported in the studies, n (%)	Decrease reported in the studies, n (%)
**Self-care maintenance**			
	Medication adherence (n=15)	4 (27)	8 (53)
	Physical activities (n=20)	7 (35)	13 (65)
	Diet control (n=14)	7 (50)	8 (57)
**Self-care monitoring**			
	Self-monitoring of signs and symptoms (n=7)	4 (57)	3 (43)
**Self-care management**			
	Consultations with health care providers (n=23)	13^a^ (57)	23 (100)

^a^Replaced with telehealth.

Expanded results of self-care behaviors and technology adopted or recommended in the included articles are outlined in Multimedia Appendix 11.

### Self-care Maintenance

#### Medication Adherence

A total of 15 studies reported on medication adherence during COVID-19 [18-32]. Increase in medication adherence among participants trying to maintain good glycemic control during the pandemic was reported in 27% (4/15) [18,19,23,24] of the studies. Participants had difficulties in sourcing medication due to supply shortages in 53% (8/15) of these studies [20-22,25,27,29,30,32], with people in rural areas faring worse [29].

In one study, the participants reported lower levels of medication adherence due to store closures, fears of contracting the virus if they went outside, and difficulties in purchasing caused by financial constraints due to job losses [30]. A few studies [25,28,32] reported on difficulties in obtaining prescription renewals among participants due to cancellation of physical consultations, and telehealth was used for prescription renewals.

#### PA

A total of 20 studies reported on PA during the pandemic [18,20,24,26-29,33-45], and 35% (7/20) of these studies [28,33,38,40-43] reported on continual or increase in PA among the participants in their studies. Disruptions in routines, fear of going outdoors, lack of motivation, as well as increased anxiety and stress during COVID-19 led to the decline in PA in 65% (13/20) of these studies [18,20,24,26,27,29,34-37,39,44,45].

The participants in a study that measured PA exclusively [34] reported that 71.5% of Brazilian adults were not meeting the minimum PA recommendations. The study revealed that increasing age and multimorbidity had a positive association with increased sedentary risk during the pandemic.

#### Diet Control

A total of 14 studies reported on dietary habits during the pandemic [18-20,24,27,29,30,33,35,37,38,41,45,46], and 50% (7/14) of these studies [19,20,33,37,38,41,46] reported improved diet management among their participants. Reasons were of increased time availability, with more meals being prepared at home and lockdown restrictions limiting takeaway consumptions among participants.

There was an association between better diet control and improved glycemic control reported among participants [19,33,37,38,41,46]. Poor diet control was found in 57% (8/14) of these studies [18,20,24,27,29,30,35,45], with participants reducing their consumption of fresh fruit and vegetables due to unavailability, as there were with disruptions to supplies and reduced frequency in shopping to minimize exposure during the pandemic [25,28,32]. Increase in unhealthy food consumption was due to multiple factors such as more sedentary time at home and changes in mood including lack of motivation, boredom, increased anxiety, and stress.

### Self-care Monitoring: Self-monitoring of Signs and Symptoms

In total, 7 studies reported on participants monitoring their diabetes mellitus [18,19,24,33,37,38,41]. Regular compliance or increase in monitoring of blood glucose levels during the pandemic among participants was found in 57% (4/7) of these studies [18,19,24,37]. Decline in monitoring of blood glucose levels among participants were due to difficulties in sourcing testing strips and lack of knowledge barriers in 43% (3/7) of studies [33,38,41].

### Self-care Management: Consultations With Health Care Providers

All 23 studies that examined access to health care providers during the pandemic found disruptions to health care services, with postponement or cancellation of consultations noted among their participants [19-25,27-32,40,42,44,45,47-52]. In the 23 studies that reported on access to health care providers, 57% (13/23) of these studies [19,20,23,24,29,31,32,36,42,46,​^49^,^52^,^53^] revealed participants used telehealth services in place of physical consultations with their health care team. Moreover, 13% (3/23) of these studies [21,40,45] found that difficulties in accessing health care services during the pandemic led to issues with glycemic control among the individuals. The results of technology used in the included studies are illustrated in Table 3.

**Table 3:** Use of technology for self-care of chronic conditions in the included studies (n=26).

Technology reported in the studies	Values, n (%)
Telehealth used during COVID-19 among participants	13 (50)
Other digital technology (television, social media apps, smartphone apps, web-based digital health tools, web-based platforms, and web browsing)	13 (50)
Role of telehealth discussed and recommended	20 (77)

### Technology: Technological Aids Used

In total, 26 studies discussed the role of technology during COVID-19 to support individuals’ self-care of chronic conditions [19-29,31,32,35,36,40,42,43,45,46,48-53], and 50% (13/26) of the studies [19,20,23,24,29,31,32,36,42,46,49,52,53] reported on the use of telehealth, due to in-person consultations having been replaced with telephone or video consultations. Participants used telehealth for prescription renewals, test results discussion, or simple follow-ups. Moreover, 50% (13/26) of the participants in these studies [19,20,23,24,29,31,32,​^36^,^42^,^46^,^49^,^52^,^53^] expressed that telehealth allowed continuity of care for them during the pandemic, that the support helped them maintain their self-care behaviors, and that they would continue using it in the future.

The use of television, social media apps, smartphone apps, web-based digital health tools, web-based platforms, and web browsing was found in 50% (13/26) of these studies [19,20,26-29,32,35,36,43,48,52,53]. One study [36] reported people living with diabetes and liver disease were the highest users of social media, while video consultations were mostly used by people living with chronic liver diseases and neurological conditions in their population sample.

The role of telehealth was discussed and recommended in the future delivery of health care services in 77% (20/26) of these studies [19-22,24-26,29,31,32,36,40,42,45,46,49-53], especially for people living in rural areas [46,50]. According to one study [48], effective intervention strategies are needed to improve digital literacy among elderly people living with chronic conditions to facilitate their participation and presence in digital health.

Telehealth was the most used technology, followed by social media apps (Facebook), messaging apps (WhatsApp, Messenger, and WeChat), web-based platforms for education and exercise (YouTube and web-based exercise platforms), and web browsing (Google).

Multimedia Appendix 12 provides details on the types of technology used and recommended in the included articles.

## Discussion

### Principal Findings

To our knowledge, this is the first systematic review of changes in self-care behaviors in people with chronic conditions and the technological aids they adopted in managing their conditions during COVID-19.

The purpose of this systematic review was to analyze the existing literature on how self-care behaviors have changed during COVID-19, and the range of technology adopted by people with chronic conditions in managing their conditions during the pandemic. Our results indicate that the lives of people with chronic conditions were altered by the course of measures imposed to contain the spread of COVID-19, with disruptions to their daily routines challenging their self-care behaviors. The lockdown resulted in both favorable and unfavorable changes in self-care behaviors, which could have short- and long-term effects on health.

Positive self-care behaviors that resulted from the lockdown were found among individuals motivated to keep good glycemic control, and those who maintained or increased their medication adherence during COVID-19. Improved diet control resulted from an increase in home cooking and less consumption of takeaways. Cancelled physical consultations were replaced with telehealth to allow continuity of health care services.

Negative self-care behaviors that resulted from the lockdown were from fluctuations in medical supplies, difficulties in sourcing prescriptions, and financial constraints impacting medication adherence. Reduction in fresh produce consumption due to supply issues and lack of motivation led to poor diet control. Significant decline in PA and increased sedentariness were found among participants in most studies during lockdown. In-person visits for routine consultations were postponed or cancelled. Access to health care services was facilitated by telehealth through phone or video consultations to allow continuity of care during the pandemic. However, in some developing counties, proactive contact with people with chronic diseases during the pandemic with telehealth was rare [30,33,34,41,44,47].

The role of technology in the home setting to manage chronic conditions remains low with telehealth being the most frequently used technology during COVID-19, followed by internet browsing, social media platforms, and messaging apps. There is a lack of studies focusing on the effects of eHealth, mobile health, and health apps in the delivery of health care services or management of self-care during COVID-19; this then presents an opportunity for future research in this area.

### Strengths and Limitations

This review has several strengths. We developed and followed a rigorous and predefined protocol that was registered with the International Prospective Register of Systematic Reviews (PROSPERO) database at the beginning of the study. To ensure sensitivity and specificity, we developed an extensive search strategy of literature with the help of a clinical librarian. Eligibility criteria were objectively stated and applied in the screening of each article by 3 independent reviewers, and there was substantial agreement with the full text screening results.

There were some limitations in the review, as only articles published in English were included, and we did not have access to studies in other languages. The use of validated instruments to measure the effect of COVID-19 public health measures on self-care behaviors across all studies was limited. The review focused only on self-care behaviors undertaken by people living with chronic conditions, leaving out the caregiver’s role in managing self-care. Only 4 studies used qualitative approaches, and there is a need to increase the use of qualitative methodology in self-care research to gain more insights or context on the circumstances involved.

It is important to note that search of databases consisted of keywords such as “chronic conditions” and “multimorbidity” and not the exact diagnosis terms, which may result in excluding articles that use exact diagnosis terms (eg, “diabetes” and “cardiovascular”). The majority of studies examined technology that will enable communications with people with chronic conditions and their health care provider. However, the use of other digital tools that help in monitoring and providing aid in managing their conditions was limited. There is a need for further research on the use of other types of technology and how it was used in the management of self-care in the home environment.

### Comparison With Existing Literature

A recent review focusing on lifestyle changes during COVID-19 [54] found increased consumption of unhealthy food and decline in PA across various population groups. The use of telehealth was widespread, and the review proposed the use of virtual networks in the future delivery of health care services, which is in line with our findings.

Kendzerska et al [12] focused on chronic disease management in the primary and specialty care settings. There were concerns that medicine shortages during COVID-19 and the decline in physical activity found among people with chronic conditions could exacerbate their conditions. The implementation of telemedicine during the pandemic outbreak has been associated with many barriers especially among elderly patients with digital literacy being a common issue, as we observed in our included study [12].

Most reviews on people with chronic conditions during the pandemic analyzed the prevalence of chronic conditions in patients diagnosed with COVID-19 and the adverse clinical outcomes associated with the population group. Increased age and underlying chronic conditions were the strongest predicators of longer hospitalizations or mortality rates among patients diagnosed with COVID-19 [55-57].

In our review, the lived experiences of people with chronic conditions and how they managed their self-care behaviors during the pandemic were examined; we also analyzed the role of digital technology in facilitating them.

### Implications

The COVID-19 experience provides an opportunity to rethink what worked and what did not during the pandemic, and to better prepare for future pandemics or health threats.

#### Key Implications 1: Self-care Behaviors

The evidence from this review shows that certain groups of people with chronic conditions managed to improve or continue with their self-care behaviors amid the pandemic while others struggled to manage them. There is a need for in-depth study on how certain population groups were able to maintain this behavior and the coping strategies they adopted.

These findings can be drawn upon to enhance current self-care interventions to further empower and support these individuals in sustaining their self-care capabilities. It can help individuals to independently cope with self-care behaviors and maintain positive health outcomes, particularly in circumstances when health care resources are redirected toward infectious disease control.

Further research is required on why certain individuals failed to engage in effective self-care behaviors during COVID-19. The factors or barriers that affected this adverse behavior needs to be investigated. The findings can be used to develop successful strategies or interventions to reinforce better health-promoting behaviors and increased adherence to self-care behaviors among this population group. The experiences of COVID-19 have shown how integral self-care is in chronic conditions management. The health care system should use this opportunity to work on a systemic approach to tackle health inequities and incorporate self-care management into the fabric of health care services. Health care professionals also need to evaluate how able individuals are in understanding the information on self-care behaviors provided to them, and their capability to engage in self-care independently.

Hence, health care professionals should tailor self-care advice and plan at the individuals’ level of understanding, their capacity, and the context they are in, so that their actions are effective and sustainable for a longer period of time.

#### Key Implications 2: Digital Technology Adoption

The COVID-19 outbreak has changed the conversation on digital interventions in health care services. The rapid adoption of telehealth, as well as the tidal of acceptance by individuals and health care providers in the delivery of health care services, has led to telehealth emerging as the silver lining of the pandemic. It has re-envisioned chronic care management and opened opportunities of using evidence-based digital health interventions that can promote and support self-care capabilities among people with chronic conditions both now and in any future public health crisis.

The reassignment of the health care resources during COVID-19 on prioritizing communicable disease care severely disrupted chronic care management with cancelled or postponed health care services. This led to a backlog of routine services and a decline in screening and preventive care that could later exacerbate health risks and strain the health care system. Care pathways need to be reconfigured to allow new models of health care to treat both communicable and chronic diseases continuously. Embedding and accelerating digital changes in chronic care management can instigate individuals and health care providers to work on solutions that allow chronic care management to be maintained alongside communicable diseases in future pandemics or health threats. Future digital health interventions should consider the influence of family and friends in the health management of people with chronic condition. They play a major role in supporting or assisting individuals with making daily decisions about medications and symptoms management, helping coordinate health care services and facilitate healthy behavior changes. It is important that newer digital health interventions recognize and provide digital solutions for all members of the individual care team for optimal health outcomes.

There is a lack of research on vulnerable population groups (ie, elderly, indigenous communities, and disability groups) who are at a greater risk of contracting COVID-19 and the associated population health implications. These population groups must be the focus of future studies, evaluating their lived experiences in managing their chronic conditions and use of technology during COVID-19. Any disparities identified in access, digital literacy, and equity should be appropriately addressed. The lessons of the pandemic should not be lost; they should be used to build new approaches in chronic self-management.

### Conclusions

This review provided insights into how people with chronic conditions managed their self-care behaviors during COVID-19, and the types of technology used during that period. In our systematic review, we found that the measures imposed to mitigate the spread of COVID-19 did have an impact on people with chronic conditions and their self-care capabilities, resulting in the decline in PA and self-monitoring of signs and symptoms, increase in unhealthy food consumption, and difficulties in medication adherence.

There are concerns that if these negative self-care behaviors are sustained postpandemic, they could lead to further health complications among people with underlying chronic conditions and burden the overstretched health care system.

The lived experiences of COVID-19 should become a catalyst for adoption of a new model for health care that is flexible to respond to both chronic and infectious diseases. It should recognize and have measures in place to support and enhance self-care capabilities among people with underlying chronic conditions during the pandemic, and for future health threats. The use of digital technology (telehealth, online platforms, and messaging apps) connected individuals to health care services and changed the way they receive care during the pandemic. This highlights the need for further research on incorporating and leveraging evidence-based digital health tools into newer models of health care. These can then aim to engage and motivate individuals toward the effective management of their self-care behaviors and facilitate continuity of health care services in any situation.

## Appendix

Multimedia Appendix 1 PRISMA (Preferred Reporting Items for Systematic Reviews and Meta-Analyses) 2020 checklist.

Multimedia Appendix 2 Search strategy.

Multimedia Appendix 3 Inclusion and exclusion criteria.

Multimedia Appendix 4 Search and screening process.

Multimedia Appendix 5 Methodological quality assessment.

Multimedia Appendix 6 Health conditions stated in the included studies.

Multimedia Appendix 7 Self-care behaviors included in the studies.

Multimedia Appendix 8 Technology stated in the included studies.

Multimedia Appendix 9 Characteristics of the included studies.

Multimedia Appendix 10 Characteristics of the included studies.

Multimedia Appendix 11 Results.

Multimedia Appendix 12 Technology used and recommended.

## Abbreviations

PA: physical activities
PRISMA: Preferred Reporting Items for Systematic Reviews and Meta-Analyses
PROSPERO: International Prospective Register of Systematic Reviews
