# Supplementary figures and images for: Self-care Behaviors and Technology Used During COVID-19: Systematic Review
Source: JMIR Hum Factors. 2022 Jun 21;9(2):e35173. doi: 10.2196/35173 (PMC9217152; doi:10.2196/35173)

**
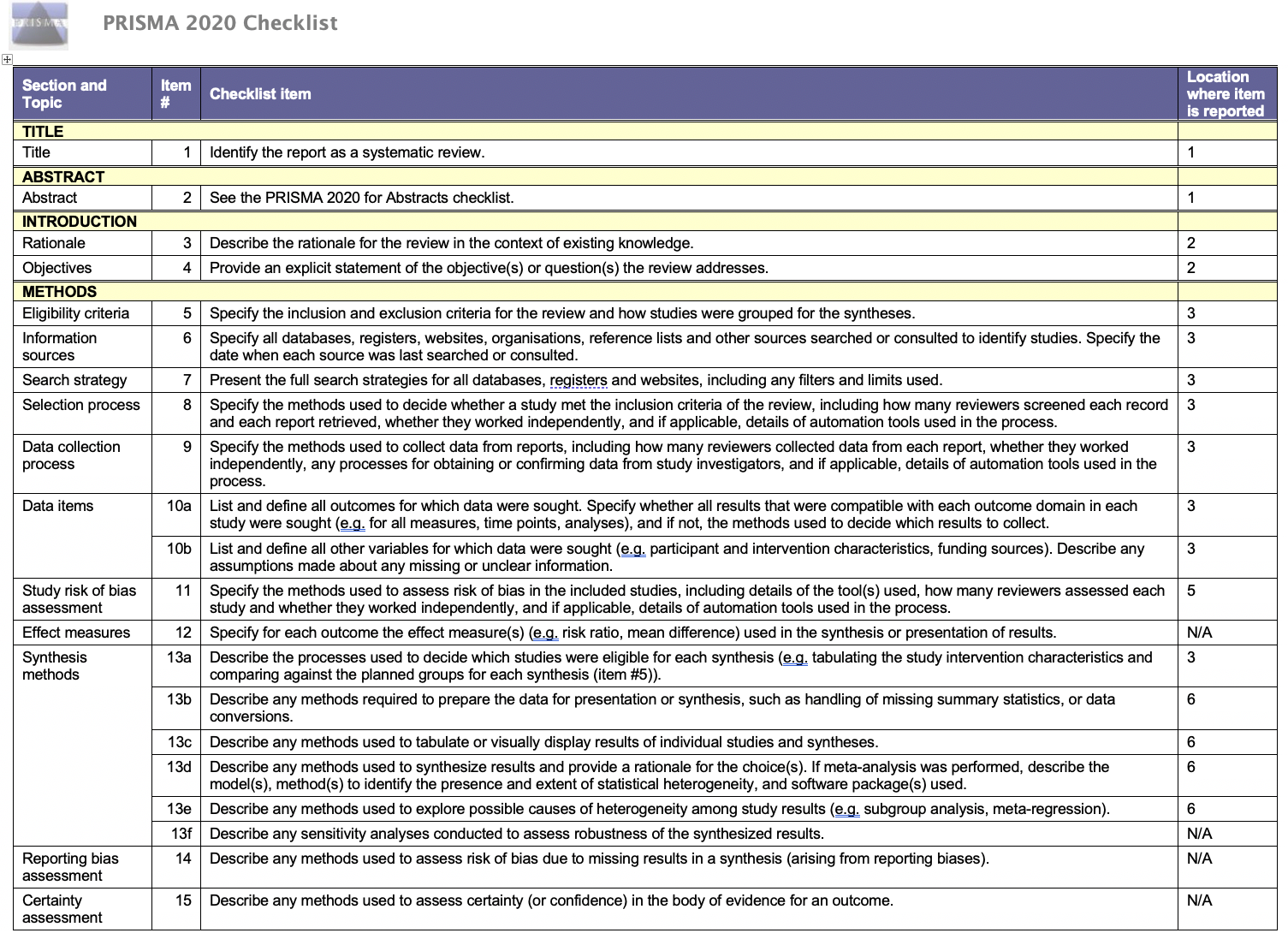
**

**
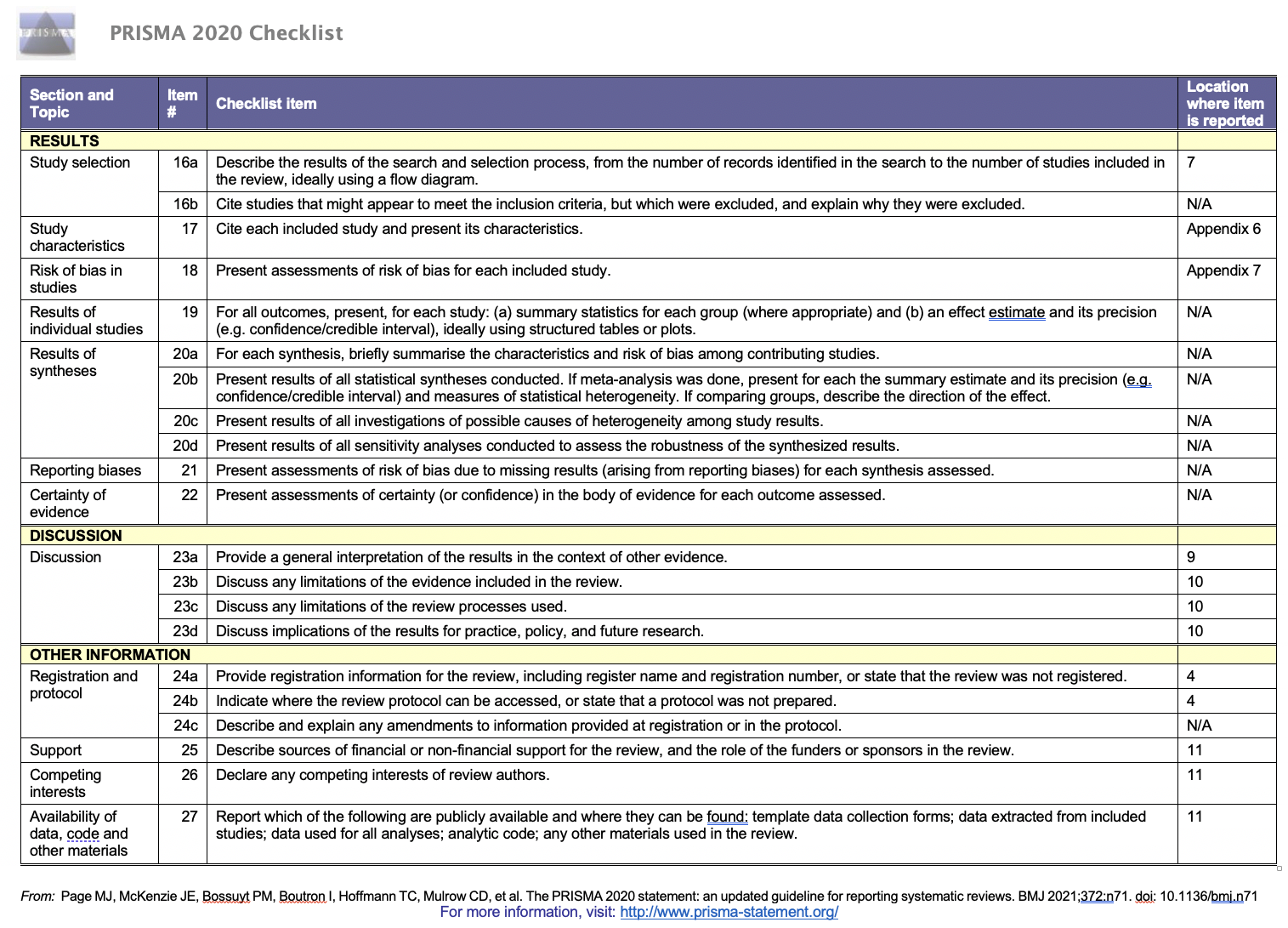
**

Supplement: Multimedia Appendix 1 [file humanfactors_v9i2e35173_app1.docx]
