# Supplementary material for: Self-care Behaviors and Technology Used During COVID-19: Systematic Review
Source: JMIR Hum Factors. 2022 Jun 21;9(2):e35173. doi: 10.2196/35173 (PMC9217152; doi:10.2196/35173)
Supplement: Multimedia Appendix 2 [file humanfactors_v9i2e35173_app2.docx]

 The search terms used to conduct the search were terms related to “chronic conditions”, “COVID-19”, “self-care” and “technology” were used in various combinations. Appropriate Boolean operators (“OR” and “AND”), proximity operations (“N” and “ADJ”) and truncation were used to combine search terms. The search was limited to English language. A search strategy was developed in line with keywords used in similar studies [61,62] along with consultations with clinical librarian and this was used across all databases.


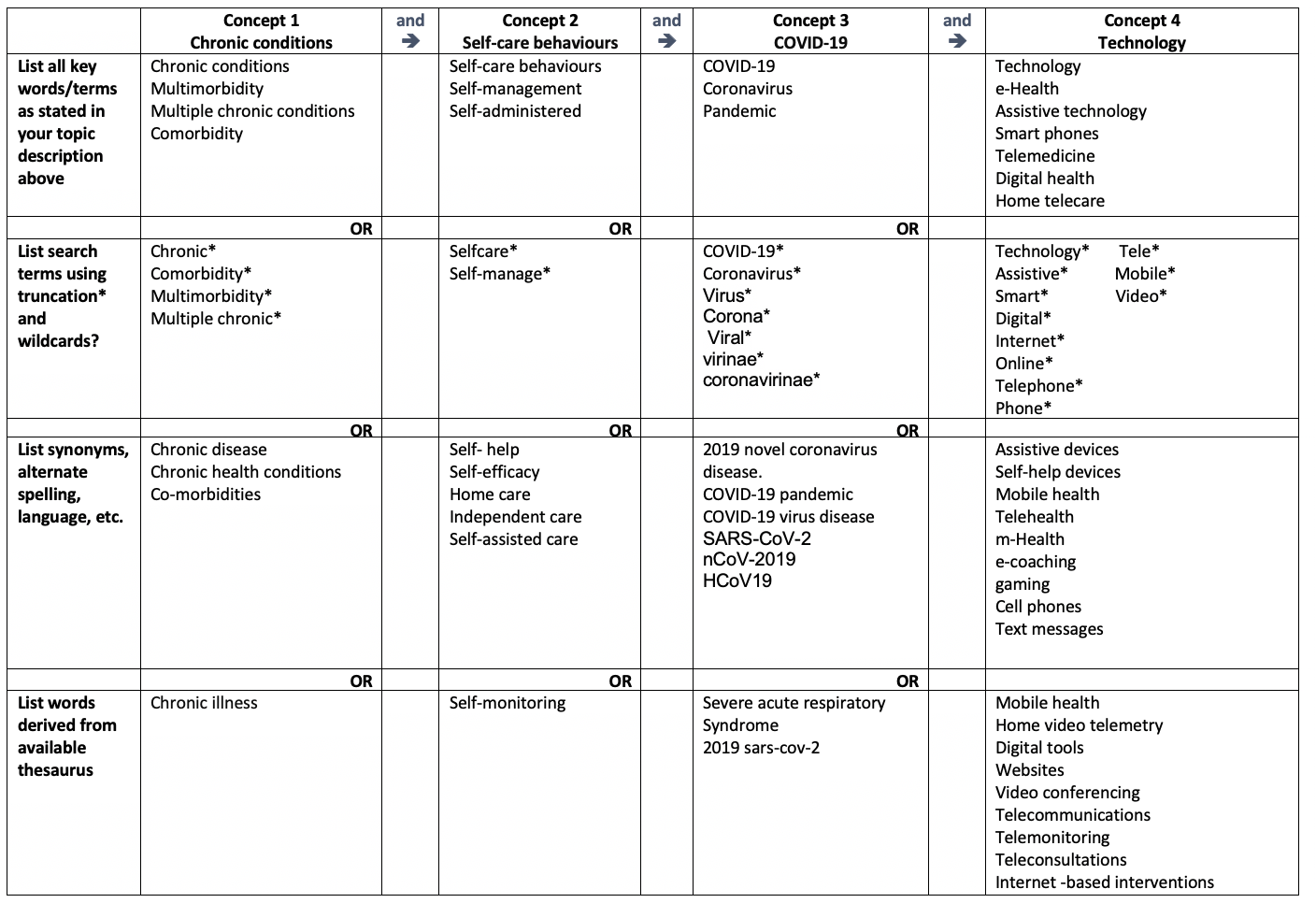


**The full electronic search strategy applied across all databases (excluding Google Scholar)**

| 1 | chronic disease/ or multiple chronic conditions/ |
| --- | --- |
| 2 | comorbidity/ or multimorbidity/ |
| 3 | chronic* or multiple chronic*or multimorbidity* or comorbidity).ti,ab. |
| 4 | self-care/ or self-administration/ or self-medication/ or self-testing/ |
| 5 | Self-Care/ or Self-Management/ |
| 6 | (self-care* or self-manage* or self-monitor*).ti,ab. |
| 7 | (self-help or self-efficacy or home care or independent care or self-assisted).ti,ab. |
| 8 | or/1-3 |
| 9 | or/4-7 |
| 10 | and/8-9 |
| 11 | exp coronavirus/ |
| 12 | ((corona* or corono*) adj1 (virus* or viral* or virinae*)).ti,ab,kw. |
| 13 | (coronavirus* or coronovirus* or coronavirinae* or Coronavirus* or Coronovirus* or Wuhan* or Hubei* or Huanan or "2019-nCoV" or 2019nCoV or nCoV2019 or "nCoV-2019" or "COVID-19" or COVID19 or "CORVID-19" or CORVID19 or "WN-CoV" or WNCoV or "HCoV-19" or HCoV19 or CoV or "2019 novel*" or Ncov or "n-cov" or "SARS-CoV-2" or "SARSCoV-2" or "SARSCoV2" or "SARS-CoV2" or SARSCov19 or "SARS-Cov19" or "SARSCov-19" or "SARS-Cov-19" or Ncovor or Ncorona* or Ncorono* or NcovWuhan* or NcovHubei* or NcovChina* or NcovChinese*).ti,ab,kw. |
| 14 | ((respiratory* adj2 (symptom* or disease* or illness* or condition*)) or "seafood market*" or "food market*") adj10 (Wuhan* or Hubei* or China* or Chinese* or Huanan*)).ti,ab,kw. |
| 15 | ((outbreak* or wildlife* or pandemic* or epidemic*) adj1 (China* or Chinese* or Huanan*)).ti,ab,kw. |
| 16 | "Severe acute respiratory syndrome*".ti,ab,kw. |
| 17 | or/11-16 |
| 18 | Digital Technology/ or Technology/ |
| 19 | Telemedicine/ |
| 20 | Internet-Based Intervention/ |
| 21 | ((internet or web or online or digital) adj3 based intervention*).ti,ab,kw. |
| 22 | (Digital health or mobile health or telehealth or ehealth or mhealth or zoom or telephone* or mobile phone* or cell phone* or text messag*).ti,ab. |
| 23 | (Video conferencing or telecommunications or telemonitoring or teleconsultations).ti,ab |
| 24 | (Home video telemetry or digital tools or gaming or m- health or e-coaching or self-help devices).ti,ab. |
| 25 | (technology* or assistive or smart* or digital or internet* or online* of tele* or mobile* or video* or telephone*).ti,ab. |
| 26 | or/18-25 |
| 27 | 10 and 17 and 26 |
| 28 | from 27 keep 1-41 |
| 29 | limit 28 to yr="2019 -Current" |
