# Supplementary material for: Self-care Behaviors and Technology Used During COVID-19: Systematic Review
Source: JMIR Hum Factors. 2022 Jun 21;9(2):e35173. doi: 10.2196/35173 (PMC9217152; doi:10.2196/35173)
Supplement: Multimedia Appendix 3 [file humanfactors_v9i2e35173_app3.docx]

| **Inclusion Criteria** | **Exclusion Criteria** |
| --- | --- |
| Articles were eligible if they focused on adults diagnosed with chronic conditions. | Articles were excluded if they focused on nonpatients (e.g. caregivers or care providers). |
| Included a quantitative and/or qualitative component. | If study was not COVID-19 related. |
| Focused on reporting self-care tasks during COVID undertaken by people diagnosed with chronic conditions. This is in line with elements of self-care activities as defined by middle-range theory of self-care of chronic illness:   1. self-care maintenance - engaging in behaviours that help maintain physical and emotional stability 2. self-care monitoring - observing changes in health status 3. self-care management – assessing changes in physical and emotional signs and symptoms and determining and what cause of action is appropriate. | If they were educational programs to improve self-management of chronic conditions. |
| Articles on studies that focused on technology used in managing self-care tasks for people with chronic conditions. | Were articles that focused on technology only or the use of technology by care providers with no outcome measures |
| Articles published December 2019 onwards. | Were protocol papers or opinion articles. |
