# Supplementary material for: Self-care Behaviors and Technology Used During COVID-19: Systematic Review
Source: JMIR Hum Factors. 2022 Jun 21;9(2):e35173. doi: 10.2196/35173 (PMC9217152; doi:10.2196/35173)
Supplement: Multimedia Appendix 4 [file humanfactors_v9i2e35173_app4.docx]

The search strategy retrieved 498 publications with 54 publications from PubMed, 14 publications from Embase, 20 publications from PsychInfo, 19 publications from CINAHL, 31 publications from Medline, 360 publications from Google Scholar. The first initial screening in Endnote identified and removed 122 duplicates. 376 Abstracts were screened against the eligibility criteria and 289 abstracts were excluded.

Search updates led to 9 publications been included in the screening. 96 publications were downloaded as full texts and screened against the eligibility criteria. 63 full text publications were found to be ineligible and excluded, leaving 33 included studies. A further 3 studies were identified by searching the reference lists of included studies. The entire screening process was concluded with 36 original research publications been included.

**Study screening**

498 publications retrieved from the database searches from 6 March to 11 March 2021 were imported into Endnote (Version X9). 122 duplicates were found within Endnote and removed. The retrieved 96 publications abstract was screened by 3 independent blind reviewers using the Rayyan software, any disagreements were resolved by consensus.

33 publications that met the inclusion criteria were downloaded and 3 articles were further included from the reference list screening. by reviewer. After full text screening, 36 publications were included in the systematic review.

**Data Extraction**

Reviewers extracted descriptive data about the characteristics of the included publications. The following characteristics were collected from:

Primary research articles

The country of study; aim of the study; population characteristics; the health conditions studied; duration of disease; technology utilised; study methodology; duration of disease; measures of self-care observed in study; sample size; data collection; data analysis and key observations of the study.

Self-care behaviours and technology used

Information regarding self-care behaviours observed in the studies and types of technological aids used for management in self-care was extracted by reviewers.
