# Supplementary material for: Self-care Behaviors and Technology Used During COVID-19: Systematic Review
Source: JMIR Hum Factors. 2022 Jun 21;9(2):e35173. doi: 10.2196/35173 (PMC9217152; doi:10.2196/35173)
Supplement: Multimedia Appendix 5 [file humanfactors_v9i2e35173_app5.docx]

The methodological assessment of the included studies was undertaken with a modified version of the McMaster Critical Appraisal Tools for Quantitative Studies and Qualitative Studies.[53]

There were significant variations in the methodological quality scores. Of the quantitative studies, 28 studies scored greater than 70% [18, 19, 22, 23, 24, 25, 26, 27, 28, 29, 30, 33, 34, 35, 37, 39, 40, 42, 43, 45, 47, 48, 49, 50, 51, 52, 60] while 4 studies scored equal to or below 70%.[36, 38, 44, 46]

Amongst the quantitative research designs, studies were scored lower due to lack of explanation on sample size dropouts, lack of statistically significant reporting in results, inappropriate statistical methods, outcome reliability, and outcome validity. Amongst the 4 studies using qualitative research methodologies, all scored greater than 70%.[ 21, 31, 32, 41]

**Methodological quality of quantitative studies based on the McMaster critical appraisal tool**


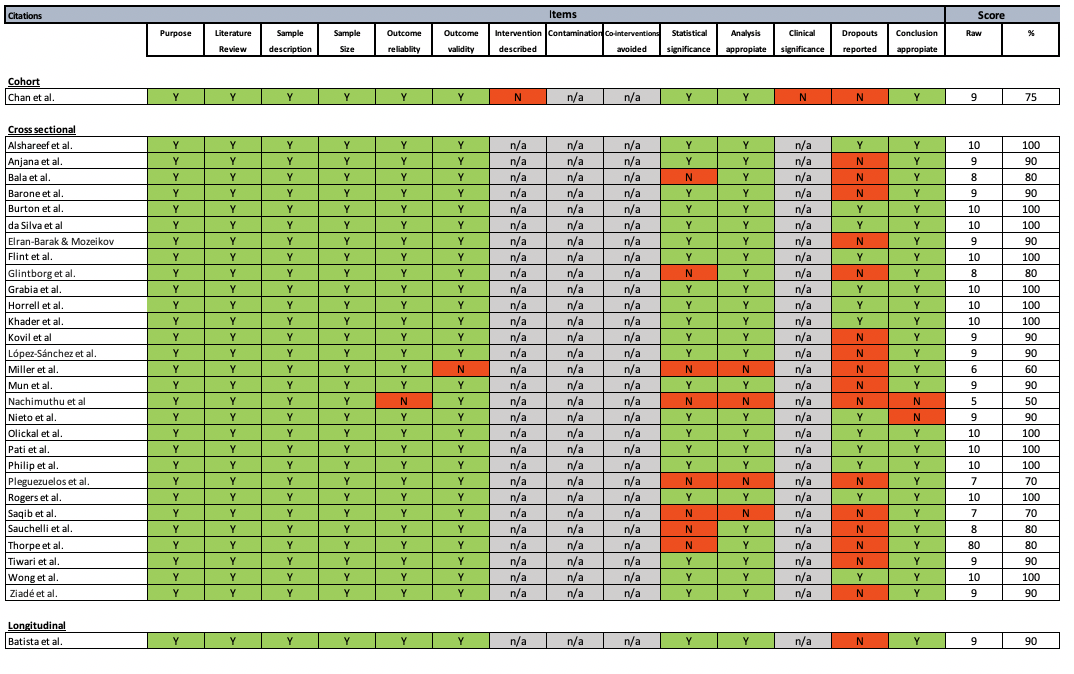


**Methodological quality of qualitative studies based on the McMaster critical appraisal tool**

**
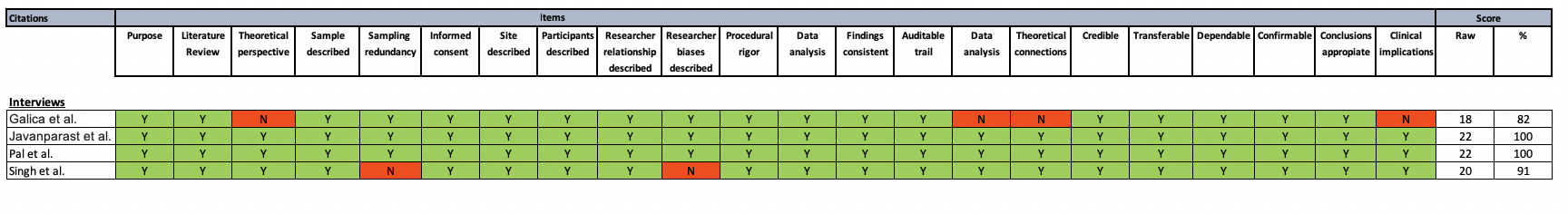
**

Y = yes (green shading); N = no (red shading).

*Source: Hong QN, Pluye P, Fàbregues S, Bartlett G, Boardman F, Cargo M, Dagenais P, Gagnon MP, Griffiths F, Nicolau B, O’Cathain A. Mixed methods appraisal tool (MMAT), version 2018. Registration of copyright. 2018;1148552:10*
