# Supplementary material for: Self-care Behaviors and Technology Used During COVID-19: Systematic Review
Source: JMIR Hum Factors. 2022 Jun 21;9(2):e35173. doi: 10.2196/35173 (PMC9217152; doi:10.2196/35173)
Supplement: Multimedia Appendix 6 [file humanfactors_v9i2e35173_app6.docx]

| **Health conditions** | **Studies** |
| --- | --- |
| Diabetes Mellitus/ Type 1/ Type 2 (N=26) | [18, 19, 20, 21, 22, 25, 26 , 28, 29, 30, 32, 33, 34, 35, 38, 40, 41, 42, 45,46, 47 48, 49, 50, 51, 60] |
| Hypertension (N=8) | [21, 25, 26, 35, 45, 46, 48, 60,] |
| Cancer (N=7) | [26, 27, 31, 32, 42,45, 60] |
| Cardiovascular diseases (N=6) | [22, 25, 26 , 27, 48, 51] |
| Chronic heart diseases (N=6) | [21, 28, 29, 38, 45, 50,] |
| Mental health (N=6) | [21, 27, 32, 46 , 50, 51] |
| Other diseases (N=6) | [26, 27, 35, 42, 43, 60] |
| Respiratory (N=6) | [24, 28, 43, 50, 51, 60] |
| Chronic kidney diseases (N=5) | [21, 28, 38, 42, 48,] |
| Chronic obstructive pulmonary disease (N=4) | [21, 43, 44, 48,] |
| Chronic lung diseases (N=3) | [29, 42, 45] |
| Chronic pain (N=2) | [37, 39] |
| Comorbidities (N=2) | [29, 33] |
| Epilepsy (N=2) | [36, 50] |
| Inflammatory rheumatic diseases (N=2) | [29, 52] |
| Non-communicable diseases (N=2) | [21, 22] |
| Obesity (N=2) | [35, 45] |
| Asthma (N=2) | [29,60] |
| Hyperlipidaemia (N=2) | [25,60] |
| Stroke (N=2) | [21,45,] |
| Autoimmune disease (N=1) | [27] |
| Cardiometabolic (N=1) | [24] |
| Chronic bronchitis (N=1) | [35] |
| Metabolic (N=1) | [27] |
| Multimorbidity (N=1) | [23] |
| Musculoskeletal issues (N=1) | [32] |
| Varicose veins (N=1) | [35] |
