# Supplementary material for: Self-care Behaviors and Technology Used During COVID-19: Systematic Review
Source: JMIR Hum Factors. 2022 Jun 21;9(2):e35173. doi: 10.2196/35173 (PMC9217152; doi:10.2196/35173)
Supplement: Multimedia Appendix 7 [file humanfactors_v9i2e35173_app7.docx]

| **Self-care maintenance:** | |
| --- | --- |
| Physical activities  (N=20) | [18, 20, 21, 26, 27, 28, 30, 33, 34, 35, 37, 38, 39, 41, 43, 44, 45, 46, 47, 48] |
| Medication adherence  (N=15) | [18, 19, 21,22, 25, 29, 33, 36, 39, 41, 43, 48, 49, 50, 52] |
| Diet control  (N=14) | [18, 19, 20, 21, 27, 30, 33, 34, 38, 40, 41, 47, 48, 49] |

| **Self-care monitoring:** | |
| --- | --- |
| Monitoring (blood glucose levels/blood pressure)  (N=7) | [18, 19, 20, 30, 33, 34, 38] |

| **Self-care management:** | |
| --- | --- |
| Consultations with health care providers  (N=23) | [19, 21, 22, 23, 25, 29, 31, 32, 33, 36, 37, 41, 42, 43, 44, 46, 47, 48, 49, 50, 51, 52, 60] |
