# Supplementary material for: Self-care Behaviors and Technology Used During COVID-19: Systematic Review
Source: JMIR Hum Factors. 2022 Jun 21;9(2):e35173. doi: 10.2196/35173 (PMC9217152; doi:10.2196/35173)
Supplement: Multimedia Appendix 8 [file humanfactors_v9i2e35173_app8.docx]

| **Technology** | |
| --- | --- |
| Telehealth (teleconsultations/ telemedicine/ remote consultation/ virtual consultation platforms/phone consultations)  (N=21) | [19, 21, 22, 24, 25, 28, 29, 32, 33, 36, 37, 40, 42, 43, 44, 47, 48, 50, 51, 52, 60] |
| Social media apps/platforms (Facebook/WhatsApp/Messaging services)  (N=8) | [19, 21, 27, 28, 36, 41, 45, 52] |
| Online platforms (YouTube, online support communities, shopping, online prescriptions services interactive websites)  (N=6) | [19, 27, 31, 41, 43, 45] |
| Television/radio  (N=2) | [45, 52] |
| Web browsing (Google search engines/ internet resources)  (N=1) | [39] |
| Telephone services (telephone services for healthcare advice)  (N=1) | [43] |
