# Supplementary material for: Self-care Behaviors and Technology Used During COVID-19: Systematic Review
Source: JMIR Hum Factors. 2022 Jun 21;9(2):e35173. doi: 10.2196/35173 (PMC9217152; doi:10.2196/35173)
Supplement: Multimedia Appendix 9 [file humanfactors_v9i2e35173_app9.docx]

| **Study author(s)/ publication year** | **Medical condition(s)** | **Self-care behaviours observed in studies are organised according to the middle range theory of self-care of chronic illness** | **Technology** | **Methodology** | **Sample size /characteristics** |
| --- | --- | --- | --- | --- | --- |
| Alshareef et al.  2020 | Type 2 diabetes mellitus and one comorbidity | Self-care maintenance: medication adherence /physical activities/diet control/  Self-care monitoring : checking blood glucose levels  Self-care management: N/A | Not stated | Semi-structured interviews, telephone, | N=394  Age: 20-60 plus  Gender: men 169, women 225  Nationality: Saudi Arabia |
| Anjana et al.  2020 | Type 2 diabetes mellitus | Self-care maintenance: medication adherence /physical activities/diet control  Self-care monitoring: monitoring blood glucose levels  Self-care management: consultations with health care providers | Telehealth  Online support platforms  Social apps/platforms | Semi-structured interviews, telephone | N=2510  Age: mean = 54 years  Gender: male 1310,  Female 1200  Nationality: India |
| Bala et al.  2021 | Diabetes mellitus | Self-care maintenance: medication adherence /physical activities/diet control  Self-care monitoring: monitoring blood glucose levels  Self-care management: N/A | Not stated | Self-reported semi-structured survey, in person | N= 108  Age: mean =56.3 years,  Gender: male 60, female 48  Nationality: India |
| Barone et al.  2020 | Diabetes mellitus and  other NCDS | Self-care maintenance: medication adherence /physical activities/diet control  Self-care monitoring: monitoring blood glucose levels  Self-care management: consultations with health care providers | Telehealth | Online semi-structured survey | N= 1562  Age: 18-80 + years,  Gender: male 371, female 1189.  Nationality: Brazil |

| Batista et al.  2020 | Multimorbidity | Self-care maintenance: sourcing medication and food supplies  Self-care monitoring: N/A  Self-care management: N/A | Not stated | Semi-structured interviews, telephone | N= 6149  Age: 50-80 years plus,  Gender: N/A  Nationality: Brazil |
| --- | --- | --- | --- | --- | --- |
| Burton et al.  2020 | Cardiometabolic, autoimmune, respiratory and  cancer | Self-care maintenance: medication adherence  Self-care monitoring: N/A  Self-care management: consultations with health care providers | Telehealth | Online semi-structured survey | N= 342  Age: mean 45-64 years  Gender: male 43, female 293, unknown 6.  Nationality: USA |
| Chan et al.  2020 | Hypertension, diabetes cardiovascular diseases and hyperlipidaemia | Self-care maintenance: medication adherence  Self-care monitoring: N/A  Self-care management: N/A | Telehealth | Semi-structured interviews, telephone | N= 765  Age: 18-64 years,  Gender: N/A  Nationality: Hong Kong |
| da Silva et al.  (2020) | Hypertension, diabetes, cardiovascular disease, cancer and other diseases | Self-care maintenance: physical activities  Self-care monitoring: N/A  Self-care management: N/A | Not stated | Semi-structured interviews, face to face | N= 249  Age: 64  Gender: male N/A ,female N/A  Nationality: Brazil |

| Elran-Barak & Mozeikov,  2020 | Mental health, metabolic,  cardiovascular, cancer,  autoimmune and other diseases | Self-care maintenance: medication adherence /physical activities/diet control/  Self-care monitoring: N/A  Self-care management: consultations with health care providers | Social media platforms  Online platform | Online semi-structured survey | N= 315  Age: 18-85 years plus  Gender: male 402, female 624  Nationality: Israel |
| --- | --- | --- | --- | --- | --- |
| Flint et al.  2020, | Diabetes, chronic respiratory diseases, chronic heart diseases and chronic kidney diseases | Self-care maintenance: medication adherence /physical activities/diet control/  Self-care monitoring: N/A  Self-care management: consultations with health care providers | Telehealth  Social media platforms | Online semi-structured survey | N= 1038  Age: mean 54.6 years,  Gender: male 402, female 624  Nationality: UK |
| Galica et al.  2020 | Breast cancer and  colon or rectal cancer | Self-care maintenance: medication adherence  Self-care monitoring: N/A  Self-care management: consultations with health care providers | Online platforms | Semi-structured interviews, telephone | N= 30  Age: mean = 72.1 years,  Gender: male 13, female 17  Nationality: Canada |
| Glintborg et al.  2021 | Inflammatory rheumatic diseases, associated comorbidities, lung diseases,  asthma, diabetes,  heart disease, cancer and  other diseases | Self-care maintenance: medication adherence /  Self-care monitoring: N/A  Self-care management: consultations with health care providers | Telehealth | Online semi-structured survey | N= 12789  Age: 39-80 plus years,  Gender: male 4423, female 8366  Nationality: Denmark |

| Grabia et al. 2020 | Diabetes mellitus | Self-care maintenance: medication adherence /physical activities/diet control/  Self-care monitoring: monitoring blood glucose levels  Self-care management: N/A | Not stated | Online semi-structured survey | N= 124  Age: mean 23 years,  Gender: male 21, female 103  Nationality: Poland |
| --- | --- | --- | --- | --- | --- |
| Horrell et al.  2021 | Hypertension hyperlipidemia asthma, migraine, cancer, Type 2 diabetes, respiratory conditions | Self-care maintenance: medication adherence  Self-care monitoring: N/A  Self-care management: consultations with health care providers | Telehealth | Online semi-structured survey | N= 2210  Age: mean 55+  Gender: male, 424,female 1781, other 5.  Nationality: USA |
| Javanparast et al.  2021, | 2 or more chronic conditions (diabetes, cancer, musculoskeletal issues and mental health) | Self-care maintenance: medication adherence  Self-care monitoring: N/A  Self-care management: consultations with health care providers | Telehealth | Semi-structured interviews, telephone | N= 30  Age: 54 and 88  years,  Gender: male 13 female 17  Nationality: Australia |
| Khader et al.  2020  India | Type 1, type 2 and gestational diabetes and comorbidities | Self-care maintenance: medication adherence /physical activities/diet control  Self-care monitoring: monitoring blood glucose levels  Self-care management: consultations with health care providers | Telehealth | Online semi-structured survey | N= 1510  Age: mean = 41.6 years. Gender: male 963, female 543, other 4  Nationality: India |

| Kovil et al.  2020 | Diabetes mellitus | Self-care maintenance: medication adherence /physical activities/diet control  Self-care monitoring: monitoring blood glucose levels  Self-care management: N/A | Not stated | Online semi-structured survey | N= 343  Age: mean =55 years  Gender: male 233, female 110  Nationality: India |
| --- | --- | --- | --- | --- | --- |
| López-Sánchez et al,  2021 | Obesity, hypertension, varicose veins of lower extremities, chronic bronchitis, diabetes type 2 and other diseases | Self-care maintenance: physical activities  Self-care monitoring: N/A  Self-care management: N/A | Not stated | Online semi-structured survey | N= 163  Age: 18-64  years,  Gender: male 47, female 113  Nationality: Spain |
| Miller et al.  2020 | Epilepsy | Self-care maintenance: medication adherence  Self-care monitoring:  Self-care management: consultations with health care providers | Telehealth  Social apps/platforms | Online semi-structured survey | N= 94  Age: mean = 36 years,  Gender: male 47, female 47  Nationality: USA |
| Mun et al. 2021 | Chronic pain | Self-care maintenance: medication adherence /physical activities  Self-care monitoring: N/A  Self-care management: consultations with health care providers | Telehealth | Online semi-structured survey | N= 14535  Age: mean = 41.7 years,  Gender: male 498, female 968, other 7  Nationality: USA |

| Nachimuthu et al.  2020 | Diabetes, kidney disease and heart disease | Self-care maintenance: medication adherence /physical activities/diet control/  Self-care monitoring: monitoring blood glucose levels  Self-care management: N/A | Not stated | Online semi-structured survey | N= 100  Age: mean = 65 + years,  Gender: male 54, female 46  Nationality: India |
| --- | --- | --- | --- | --- | --- |
| Nieto et al.  2020 | Chronic pain: musculoskeletal, headache and orofacial, neuropathic visceral, cancer,  post-surgical/post-traumatic | Self-care maintenance: medication adherence /physical activities/diet control/  Self-care monitoring: N/A  Self-care management: N/A | Web browsing | Online semi-structured survey | N= 502  Age: 18-89 years,  Gender: male 60, female 442  Nationality: Spain |
| Olickal et al.  2020 | Diabetes mellitus | Self-care maintenance: medication adherence  Self-care monitoring: monitoring blood glucose levels  Self-care management: consultations with health care providers | Telehealth | Semi-structured interviews, telephone | N= 350  Age: mean = 57 years,  Gender: male 274, female 76  Nationality: India |
| Pal et al.  2021 | Type 1 diabetes mellitus | Self-care maintenance: medication adherence /physical activities/diet control/  Self-care monitoring: monitoring blood glucose levels  Self-care management: consultations with health care providers | Online platforms  Social platforms | Semi-structured interviews, telephone | N= 30  Age: mean = 22.8 years,  Gender:  male:female 8:7  Nationality: India |

| Pati et al.  2021 | Diabetes, cancer, chronic lung diseases, chronic heart disease, chronic kidney disease and other diseases | Self-care maintenance: medication adherence /physical activities/diet control/  Self-care monitoring: monitoring blood glucose levels and blood pressure  Self-care management: consultations with health care providers | Telehealth | Semi structured interview, face to face / online | N= 600  Age: mean = 55 years,  Gender: male 295, female 305  Nationality: India |
| --- | --- | --- | --- | --- | --- |
| Philip et al.  2020 | Long-term respiratory conditions, chronic obstructive pulmonary diseases, bronchiectasis, interstitial lung disease and other diseases. | Self-care maintenance: medication adherence /physical activities  Self-care monitoring: N/A  Self-care management: consultations with health care providers | Telehealth  Telephone health advice services  Online platforms | Online semi-structured survey | N= 9515  Age: 17-80 plus years  Gender: male 2953, female 6562  Nationality: UK |
| Pleguezuelos et al.  2020 | Chronic obstructive pulmonary disease | Self-care maintenance: medication adherence /physical activities  Self-care monitoring: N/A  Self-care management: consultations with health care providers | Telehealth | Semi-structured interviews, telephone | N= 100  Age: mean = 68 years,  Gender: male 76, female 24  Nationality: Spain |
| Rogers et al.  2020 | Type 2 diabetes, lung disease, cancer, stroke, heart diseases, obesity and hypertension | Self-care maintenance: physical activities/ diet control( sourcing food supplies)  Self-care monitoring: N/A  Self-care management: N/A | Online platforms  Social apps/platforms Television | Online semi-structured survey | N= 9190  Age: 35 and 69 years, Gender: male 1914, female 7143, other 133  Nationality: UK |

| Saqib et al.  2020 | Diabetes, mental health, hypertension and more than one chronic  condition, | Self-care maintenance: medication adherence /physical activities  Self-care monitoring: regular testing  Self-care management: consultations with health care providers | Not stated | Online semi-structured survey | N= 181  Age: 18- 55+ years  Gender: male 109, female 72  Nationality: Pakistan |
| --- | --- | --- | --- | --- | --- |
| Sauchelli et al.  2021 | Type 1 and type 2 diabetes | Self-care maintenance: medication adherence /physical activities/diet control/  Self-care monitoring: monitoring blood glucose levels  Self-care management: consultations with health care providers | Telehealth | Online semi-structured survey | N= 773  Age: mean = 47.9 years,  Gender: male 249, female 516, other 4  Nationality: UK |
| Singh et al.  2021 | Diabetes mellitus, hypertension, cardiovascular disease chronic kidney disease and chronic obstructive pulmonary disease | Self-care maintenance: medication adherence /  physical activities/diet control/  Self-care monitoring: monitoring blood glucose levels  Self-care management: consultations with health care providers | Telehealth | Semi-structured interviews, telephone | N= 1734  Age: mean =57.8 years,  Gender: male 861, female 873  Nationality: India |
| Singh et al.  2021 | Diabetes mellitus, hypertension, heart disease, chronic kidney disease, stroke, chronic obstructive pulmonary disease, anxiety/ depression | Self-care maintenance: medication adherence /physical activities/diet control/  Self-care monitoring: N/A  Self-care management: consultations with health care providers | Telehealth  Social apps/platforms | Semi-structured interviews, telephone | N= 41  Age: mean = 56.3  years,  Gender: male 25, female 16. Nationality: India |
| Tiwari et al.  2020 | Type 2 diabetes | Self-care maintenance: medication adherence /physical activities/diet control/  Self-care monitoring: monitoring blood glucose levels  Self-care management: consultations with health care providers | Not stated | Semi-structured interviews, face to face | N= 1406  Age: 18 + years,  Gender: male N/A, female N/A  Nationality: India |
| Thorpe et al.  2021 | Epilepsy, diabetes, heart conditions, respiratory conditions and mental health | Self-care maintenance: medication adherence /physical activities  Self-care monitoring: N/A  Self-care management: consultations with health care providers | Telehealth | Online semi-structured survey | N= 463  Age: N/A  Gender: male 217, female 246  Nationality: UK |
| Wong et al.  2020 | Diabetes, cardiovascular and respiratory conditions, depression, and anxiety disorders | Self-care maintenance: N/A  Self-care monitoring: N/A  Self-care management: consultations with health care providers | Telehealth | Semi-structured survey; Part 1- face to face, Part 2- telephone | N= 583  Age: mean = 70.9 years,  Gender: male 160, female 423  Nationality: Hong Kong |
| Ziadé et al.  2020 | Chronic rheumatic diseases | Self-care maintenance: medication adherence  Self-care monitoring: N/A  Self-care management: consultations with health care providers | Telehealth  Social media platforms  Television  Radio | Online semi-structured survey | N= 2163  Age: mean = 40 years,  Gender: male 217, female 1564  Nationality: 15 Arab countries |
