# Supplementary material for: Self-care Behaviors and Technology Used During COVID-19: Systematic Review
Source: JMIR Hum Factors. 2022 Jun 21;9(2):e35173. doi: 10.2196/35173 (PMC9217152; doi:10.2196/35173)
Supplement: Multimedia Appendix 10 [file humanfactors_v9i2e35173_app10.docx]

### Multimedia Appendix 10. Characteristics of the included articles.

| **Nationality** | Saudi Arabia | | India | | India | | Brazil | | | Brazil | | USA | | | Hong Kong | | | Brazil | | Israel | | | UK | | Canada | | Denmark | |  |
| --- | --- | --- | --- | --- | --- | --- | --- | --- | --- | --- | --- | --- | --- | --- | --- | --- | --- | --- | --- | --- | --- | --- | --- | --- | --- | --- | --- | --- | --- |
| **Gender (male:female)** | 169:225 | | 1310:1200 | | 60:48 | | 371:1189 | | | N/A | | 43:293,  Unknown: 6 | | | N/A | | | N/A | | 402:624 | | | 402:624 | | 13:17 | | 4423:8366 | |  |
| **Age (years)** | 20-60+ | | 54 | | 56.3 | | 18-80+ | | | 50-80+ | | 45-64 | | | 18-64 | | | 64 | | 18-85+ | | | 54.6 | | 72.1 | | 39-80+ | |  |
| **Sample size** | 394 | | 2510 | | 108 | | 1562 | | | 6149 | | 342 | | | 765 | | | 249 | | 315 | | | 1038 | | 30 | | 12789 | |  |
| **Technology** | Not stated | | Telehealth  Online support platforms  Social apps/platforms | | Not stated | | Telehealth | | | Not stated | | Telehealth | | | Telehealth | | | Not stated | | Social media platforms  Online platform | | | Telehealth  Social media platforms | | Online platforms | | Telehealth | |  |
| **Medical condition(s)** | Type 2 diabetes mellitus and one comorbidity | | Type 2 diabetes mellitus | | Diabetes mellitus | | Diabetes mellitus  and other NCDS | | | Multimorbidity | | Cardiometabolic, autoimmune, respiratory and  cancer | | | Hypertension, diabetes cardiovascular diseases and hyperlipidaemia | | | Hypertension, diabetes, cardiovascular disease, cancer and other diseases | | Mental health, metabolic,  cardiovascular, cancer,  autoimmune and other diseases | | | Diabetes, chronic respiratory diseases, chronic heart diseases and chronic kidney diseases | | Breast cancer and  colon or rectal cancer | | Inflammatory rheumatic diseases, associated comorbidities, lung diseases,  asthma, diabetes,  heart disease, cancer and  other diseases | |  |
| **Year** | 2020 | | 2020 | | 2021 | | 2020 | | | 2020 | | 2020 | | | 2020 | | | 2020 | | 2020 | | | 2020 | | 2020 | | 2021 | |  |
| **Study author(s)** | Alshareef et al. | | Anjana et al. | | Bala et al. | | Barone et al. | | | Batista et al. | | Burton et al. | | | Chan et al. | | | da Silva et al. | | Elran-Barak & Mozeikov | | | Flint et al. | | Galica et al. | | Glintborg et al. | |  |
| **Nationality** | Poland | USA | | Australia | | | | India | | | India | | | Spain | | | USA | | | | USA | India | | Spain | | India | India | India | |
| **Gender (male:female)** | 21:103 | 424:1781 | | 13:17 | | | | 963:543,  Unknown: 4 | | | 233:110 | | | 47:113 | | | 47:47 | | | | 498:968,  Unknown: 7 | 54:46 | | 60:442 | | 274:76 | 8:7 (ratio) | 295:305 | |
| **Age (years)** | 23 | 55+ | | 54 & 88 | | | | 41.6 | | | 55 | | | 18-64 | | | 36 | | | | 41.7 | 65+ | | 18-89 | | 57 | 22.8 | 55 | |
| **Sample size** | 124 | 2210 | | 30 | | | | 1510 | | | 343 | | | 163 | | | 94 | | | | 14535 | 100 | | 502 | | 350 | 30 | 600 | |
| **Technology** | Not stated | Telehealth | | Telehealth | | | | Telehealth | | | Not stated | | | Not stated | | | Telehealth  Social apps/platforms | | | | Telehealth | Not stated | | Web browsing | | Telehealth | Online platforms  Social platforms | Telehealth | |
| **Medical condition(s)** | Diabetes mellitus | Hypertension hyperlipidemia asthma, migraine, cancer, Type 2 diabetes, respiratory conditions | | 2 or more chronic conditions (diabetes, cancer, musculoskeletal issues and mental health) | | | | Type 1, type 2 and gestational diabetes and comorbidities | | | Diabetes mellitus | | | Obesity, hypertension, varicose veins of lower extremities, chronic bronchitis, diabetes type 2 and other diseases | | | Epilepsy | | | | Chronic pain | Diabetes, kidney disease and heart disease | | Chronic pain: musculoskeletal, headache and orofacial, neuropathic visceral, cancer,  post-surgical/post-traumatic | | Diabetes mellitus | Type 1 diabetes mellitus | Diabetes, cancer, chronic lung diseases, chronic heart disease, chronic kidney disease and other diseases | |
| **Year** | 2020 | 2021 | | 2021 | | | | 2020 | | | 2020 | | | 2021 | | | 2020 | | | | 2021 | 2020 | | 2020 | | 2020 | 2021 | 2021 | |
| **Study author(s)** | Grabia et al. | Horrell et al. | | Javanparast et al. | | | | Khader et al. | | | Kovil et al. | | | López-Sánchez et al. | | | Miller et al. | | | | Mun et al. | Nachimuthu et al. | | Nieto et al. | | Olickal et al. | Pal et al. | Pati et al. | |
| **Nationality** | UK | | | Spain | | UK | | | Pakistan | | | | UK | | | India | | | India | | | India | | UK | Hong Kong | | 15 Arab Countries | | |
| **Gender (male:female)** | 2953:6562 | | | 76:24 | | 1914:7143, Unknown: 133 | | | 109:72 | | | | 249:516, Unknown: 4 | | | 861:873 | | | 25:16 | | | N/A | | 217:246 | 160:423 | | 217:1564 | | |
| **Age (years)** | 17-80+ | | | 68 | | 35 & 69 | | | 18-55+ | | | | 47.9 | | | 57.8 | | | 56.3 | | | 18+ | | N/A | 70.9 | | 40 | | |
| **Sample size** | 9515 | | | 100 | | 9190 | | | 181 | | | | 773 | | | 1734 | | | 41 | | | 1406 | | 463 | 583 | | 2163 | | |
| **Technology** | Telehealth  Telephone health advice services  Online platforms | | | Telehealth | | Online platforms  Social apps/platforms  Television | | | Not stated | | | | Telehealth | | | Telehealth | | | Telehealth  Social apps/platforms | | | Not stated | | Telehealth | Telehealth | | Telehealth  Social media platforms  Television  Radio | | |
| **Medical condition(s)** | Long-term respiratory conditions, chronic obstructive pulmonary diseases, bronchiectasis, interstitial lung disease and other diseases. | | | Chronic obstructive pulmonary disease | | Type 2 diabetes, lung disease, cancer, stroke, heart diseases, obesity and hypertension | | | Diabetes, mental health, hypertension and more than one chronic condition | | | | Type 1 and type 2 diabetes | | | Diabetes mellitus, hypertension, cardiovascular disease, chronic kidney disease and chronic obstructive pulmonary disease | | | Diabetes mellitus, hypertension, heart disease, chronic kidney disease, stroke, chronic obstructive pulmonary disease, anxiety/ depression | | | Type 2 diabetes | | Epilepsy, diabetes, heart conditions, respiratory conditions and mental health | Diabetes, cardiovascular and respiratory conditions, depression, and anxiety disorders | | Chronic rheumatic diseases | | |
| **Year** | 2020 | | | 2020 | | 2020 | | | 2020 | | | | 2021 | | | 2021 | | | 2021 | | | 2020 | | 2021 | 2020 | | 2020 | | |
| **Study author(s)** | Philip et al. | | | Pleguezuelos et al. | | Rogers et al. | | | Saqib et al. | | | | Sauchelli et al. | | | Singh et al. | | | Singh et al. | | | Tiwari et al. | | Thorpe et al. | Wong et al. | | Ziadé et al. | | |
