# Supplementary material for: Self-care Behaviors and Technology Used During COVID-19: Systematic Review
Source: JMIR Hum Factors. 2022 Jun 21;9(2):e35173. doi: 10.2196/35173 (PMC9217152; doi:10.2196/35173)
Supplement: Multimedia Appendix 11 [file humanfactors_v9i2e35173_app11.docx]

Results were arranged under the middle range theory of self-care of chronic illness’s key concepts; self-care maintenance (medication adherence, physical activities and diet control), self- care monitoring (self- monitoring of signs and symptoms) and self-care management (consultations with health care providers) and technology.

| **Self-care maintenance** | | |
| --- | --- | --- |
| **Medication Adherence** | **Studies** | **Results** |
| Number of studies that reported on medication adherence  (N=15) | [18, 19, 21, 22, 25, 29, 33, 36, 39, 41, 43, 48, 49, 50, 52] | 15/36 = 42% of published studies included in the review reported on medication adherence |
| Increase in medication adherence reported  (N=4) | [18, 19, 29, 33] | 4/15= 27% of studies reported an increase in medication |
| Poor adherence to medication reported  (N=8) | [22, 21, 25, 36, 41, 48, 49, 52] | 8/15 = 53% of studies reported difficulties in adhering to medication |
| **Physical Activities** | **Studies** | **Results** |
| Number of studies that reported on physical activities (N=20) | [18, 20, 21, 26, 27, 28, 30, 33, 34, 35, 37, 38, 39, 41, 43, 44, 45, 46, 47, 48] | 20/36 = 56% of published studies included in review reported on physical activities. |
| Continual or increased physical activities  (N=7) | [20, 34, 37, 38, 43, 44, 45] | 7/20 = 35% of studies reported continual or an increase in physical activities among participants. |
| Reduction in physical activities  (N=13) | [18, 21, 26, 27, 28, 30, 33, 35, 39, 41, 46, 47, 48] | 13/20 = 65% of studies reported decline in physical activities among participants. |
| **Diet control** | **Studies** | **Results** |
| Number of studies that reported on diet control (N=14) | [18, 19, 20, 21, 27, 30, 33, 34, 38, 40, 41, 47, 48, 49] | 14/36 = 39% of published studies included in the review reported on diet control |
| Improved diet management  (N=7) | [19, 20, 21, 30, 34, 38, 40, ] | 7/14 = 50% of studies reported on improved diet management among participants. |
| Poor diet control  (N=8) | [18, 21, 27, 33, 41, 47, 48, 49] | 8/14 = 57% of studies reported on poor diet control among participants |

| **Self-care monitoring** | | |
| --- | --- | --- |
| **Self-monitoring of signs and symptoms** | **Studies** | **Results** |
| Studies that reported on self- monitoring of signs and symptoms  (N=7) | [18, 19, 20, 30, 33, 34, 38] | 7/36 = 19 % of published studies included in review reported on self-monitoring of signs and symptoms |
| Continual or increase in monitoring of health status  (N =4) | [18, 19, 30, 33] | 4/7 = 57% reported compliance or increase in self-monitoring of signs and symptoms among participants |
| Decline in monitoring  (N=3) | [20, 34, 38] | 3/7 = 43% reported decline in self-monitoring of signs and symptoms among participants |

| **Self-care management** | | |
| --- | --- | --- |
| **Consultations with Health Care Providers** | **Studies** | **Results** |
| Studies that reported on consultations. (N=23) | [19, 21, 22, 23, 25, 29, 31, 32, 33, 36, 37, 41, 42, 43, 44, 46, 47, 48, 49, 50, 51, 52, 60] | 23/36 =64% of published studies included in review reported on consultations with health care providers. |
| Postponement/ cancellations of consultations.  (N=23) | [19, 21, 22, 23, 25, 29, 31, 32, 33, 36, 37, 41, 42, 43, 44, 46, 47, 48, 49, 50, 51, 52, 60] | 23/23 = 100% of studies reported postponement/ cancellations of consultations among participants. |
| In person consultations replaced with telehealth.  (N=13) | [19, 21, 24, 28, 29, 32, 33, 40, 44, 48, 50, 52, 60] | 13/23 = 57% of studies reported in person consultations were replaced with telehealth. |
| Difficulties in accessing health care services affecting glycaemia control among participants.  (N=3) | [22, 37, 47] | 3/23=13% of studies reported on participant’s glycaemic control been affected by difficulties in accessing health care services . |

| **Technology** | | |
| --- | --- | --- |
| **Technology** | **Studies** | **Results** |
| Studies that reported on technology (N=26) | [19, 21, 22, 24, 25, 27, 28, 29, 31, 32, 33, 36, 37, 39, 40, 41, 42, 43, 44, 45, 47, 48, 50, 51, 52, 60] | 26/36 = 72% of published studies included in review reported on technology |
| Telehealth was conducted via phone or virtual consultations  (N=13) | [19, 21, 24, 28, 29, 32, 33, 40, 44, 48, 50, 52, 60] | 13/26= 50% of studies reported on use of telehealth |
| Television, social media apps, smart phone apps, online digital health tools,  online platforms, web browsing  (N=13) | [19, 21, 24, 27, 31, 28, 39, 41, 43, 45, 48, 52, 60] | 13/26 = 50% of studies reported on usage of other digital technology |
| Participants expressed satisfaction in usage of telehealth and/or telehealth was recommended for continuity of care during pandemic.  (N=20) | [19, 21, 22, 24, 25, 28, 32, 33, 36, 37, 39, 40, 42, 44, 47, 48, 50, 51, 52, 60] | 20/26 =77% of studies found participants were satisfied or will continue to use telehealth, and/or the study recommended use of telehealth to allow continuity of care during rapidly evolving situations in the future. |
