# Supplementary material for: Self-care Behaviors and Technology Used During COVID-19: Systematic Review
Source: JMIR Hum Factors. 2022 Jun 21;9(2):e35173. doi: 10.2196/35173 (PMC9217152; doi:10.2196/35173)
Supplement: Multimedia Appendix 12 [file humanfactors_v9i2e35173_app12.docx]

| **Studies** | **Technology used** | **Findings** |
| --- | --- | --- |
| Anjana et al.  ( 2020) | Telehealth  Online support platforms  Social apps /platforms | - 30.6% participants used telehealth facility, of that 82% expressed satisfaction with the experience, and 58% were willing to use it in the future. - Participants used YouTube (49.1%) as online support tool for control of diabetes during lockdown, followed by Facebook (39.4%), WhatsApp (38.7%), and Google search (10.8%). - Nearly 28% of participants were interested in using an app to help with diabetes management in the future |
| Burton et al.  (2020) | Telehealth | - 74.6% of participants reported that their healthcare provider provided telehealth. - 40.7% used telehealth services during the pandemic and of that 86.8% stated they were satisfied with their experience. - 12.7% of participants( have access or plan to use telehealth) reported they will not remain using it after the pandemic. |
| Elran-Barak et al.  (2020) | Social media platforms  Online platform | - The time spend on social media and online health communities, significantly increased one month into enforcement of lockdown measures. |
| Flint et al.  (2020) | Telehealth  Social media platforms | - Telephone care was used by nearly half the participants with individuals aged 70 years or over less likely to use telephone care. - Social media usage was highest among people living with diabetes and liver disease, while virtual consultations was most likely used by people living with chronic liver diseases and neurological conditions. - Majority of participants expressed satisfaction in using the new platforms and information provided, they would consider continual use. |
| Galica et al.  (2020) | Online platforms | - Participants conducted online grocery shopping and used online platforms as Facetime and Zoom for social connections with friends and loved ones. - They found this mode of interacting tiring over time and missed physical interactions. Some participants found the online platforms challenging and instead used email or telephone to stay connected. |
| Glintborg et al.  (2021) | Telehealth | - 64% of participants were in contact with their rheumatology clinic by phone during the first 2 months of lock-down. |
| Horrell et al.  (2021) | Telehealth | - 49% of participants reported engaging in telehealth in the past 4 months of study. - Adults aged 56 years and over used telehealth were less engaged or interested in using telehealth than other participants in the study. |
| **Studies** | **Technology used** | **Findings** |
| Javanparast et al.  (2021) | Telehealth | - Telehealth was used mostly for prescription renewal, discussing test results and simple follow-ups. - Telephone was preferred over video consultations in this study, probably due to lower levels of digital literacy among older participants. - A hybrid model of telehealth and in person consultations was recommended to allow continuity, accessibility and equity in delivery of health care services. |
| Khader et al.  (2020) | Telehealth | - 7.68% of participants used virtual consultations before and this increased to 29.80% during the pandemic. - The use of telemedicine and digital tools was encouraged among people living with diabetes and NCDs. |
| Mun et al.  (2021) | Telehealth | - 29.5% of appointments were cancelled, while 24.5% had their appointments postponed. - 16% of these participants had their appointments changed to telehealth. - Telehealth and/or evidence-based online interventions was suggested for use during the pandemic to aid patients experiencing acute pain aggravation. |
| Nieto et al.  (2020) | Web browsing | - Internet resources was used by one third of participants for pain management. - Study recommends setting up of online community networks and eHealth to support people with chronic pain in uncertain situations |
| Olickal et al.  (2020) | Telehealth | - 90% of participants were willing to use telemedicine. 91% of rural area participants were more willing compared to 85% of urban participants in utilising telehealth. - 18 participants had knowledge of existing diabetes clinic’s telehealth facility, of that only 3 participants from rural areas used the services during the study. |
| Pal et al.  (2021) | Online platforms  Social platforms | - 1/30 participant used YouTube to perform yoga during lockdown. - 1/30 was in touch with diabetes educators through social platforms. |
| Philip et al.  (2020) | Telehealth  Telephone health advice services  Online platforms | - The telephone service health education and information were used by 8% of participants, while only 31% used remote access for consultations e.g. phone. - One third of participants did not access online prescription services , while over half did not view online inhaler usage educational videos. |

| **Studies** | **Technology used** | **Findings** |
| --- | --- | --- |
| Pleguezuelos et al.  (2020) | Telehealth | - The majority of patients had medical tests and/or clinical visits cancelled due to lockdowns. - Participants who used telephones for medical consultations expressed satisfaction in using the services. |
| Rogers et al.  (2020) | Online platforms  Social apps/platforms Television | - Participants watched television and used the following online services during lockdown: support groups, groceries, flute lessons through Skype, exercises classes, church meetings. - Messaging apps ( Zoom and WhatsApp) was used to connect with friends and family. |
| Singh et al.  (2021) | Telehealth | - Teleconsultations was utilised by few participants to avoid in person clinic visits. - Study recommended use of digital campaigns that will promote healthy behaviours, allow better self-management of NCS and minimised risk of COVID-19. - Health care services to be improved by using trained community health workers and applying technology-assisted medical interventions along with home monitoring devices. |
| Singh et al.  (2021) | Telehealth,  Social apps/platforms | - Some participants accessed healthcare services through teleconsultations via phone or WhatsApp. |
| Thorpe et al.  (2020) | Telehealth | - 48% of respondent’s appointments were over the telephone, while 9% were offered video consultations. - Some respondents preferred in person appointments especially for those with additional disabilities, including autism and hearing loss. - Certain respondents faced difficulties in adapting to health care services been delivered via telecommunications. - Findings suggested remote consultations can benefit specific population groups, while difficult for some groups to access it. |
| Ziadé et al.  (2020) | Telehealth  Social media platforms  Television  Radio | - 28.8% of respondents had remote contact with their rheumatologist. COVID-19 information was sourced though social media (73%), television (51%) newspaper (8%) and radio (6%) by respondents. - Attitude towards telehealth ; 98.8% stated they would accept it (50% through the internet and 48.8% through a telephone contact). - Study suggested need for reliable telehealth platform to allow continuity of care among patients. |
